# Peripheral blood neuroendocrine hormones are associated with clinical indices of sport-related concussion

**DOI:** 10.1038/s41598-019-54923-3

**Published:** 2019-12-09

**Authors:** Alex P. Di Battista, Shawn G. Rhind, Nathan Churchill, Doug Richards, David W. Lawrence, Michael G. Hutchison

**Affiliations:** 1 Faculty of Kinesiology & Physical Education, University of Toronto, Toronto, ON Canada; 2 Defence Research and Development Canada, Toronto Research Centre, Toronto, ON Canada; 3 Neuroscience Program, Keenan Research Centre for Biomedical Science of St. Michael’s Hospital, Toronto, ON Canada; 4 David L. MacIntosh Sport Medicine Clinic, Faculty of Kinesiology & Physical Education, University of Toronto, Toronto, ON Canada; 5 Keenan Research Centre for Biomedical Science of St. Michael’s Hospital, Toronto, ON Canada

**Keywords:** Neuroscience, Biomarkers

## Abstract

The purpose of this study was to evaluate the relationship between neuroendocrine hormones and clinical recovery following sport-related concussion (SRC). Ninety-five athletes (n = 56 male, n = 39 female) from a cohort of 11 interuniversity sport teams at a single institution provided blood samples; twenty six athletes with SRC were recruited 2–7 days post-injury, and 69 uninjured athletes recruited prior to the start of their competitive season. Concentrations of seven neuroendocrine hormones were quantitated in either plasma or serum by solid-phase chemiluminescent immunoassay. The Sport Concussion Assessment Tool version 5 (SCAT-5) was used to evaluate symptoms at the time of blood sampling in all athletes. Multivariate partial least squares (PLS) analyses were used to evaluate the relationship between blood hormone concentrations and both (1) time to physician medical clearance and (2) initial symptom burden. A negative relationship was observed between time to medical clearance and both dehydroepiandrosterone sulfate (DHEA-S) and progesterone; a positive relationship was found between time to medical clearance and prolactin. Cognitive, somatic, fatigue and emotion symptom clusters were associated with distinct neuroendocrine signatures. Perturbations to the neuroendocrine system in athletes following SRC may contribute to initial symptom burden and longer recovery times.

## Introduction

Recovery from sport-related concussion (SRC) remains a challenging process for both patients and clinicians. While recovery most frequently occurs within 1–3 weeks of injury, a subset of individuals may take months before medical clearance return-to-play (RTP)^1–3^. The current standard of care following SRC includes an initial period of rest followed by graded stages of physical activity until full sport participation is reached^2^. This process relies heavily on self-reported symptoms that span impairments to cognition, balance, vision, physical and emotional health, as well as fatigue and sleep disturbances^2,4,5^. As symptom reporting and recovery time following SRC are inexorably tied to the underlying pathobiology of injury, it is only through a greater understanding of this relationship that improvements to treatment will arise.

Evidence from advanced neuroimaging has shown that cortical structures such as the parietal and temporal lobes can be affected after mild traumatic brain injury (mTBI)^6,7^. However, subcortical structures including those belonging to the limbic system and brainstem may also be vulnerable, as this region is considered the fulcrum of force vectors bearing the maximal rotational forces during injury^8,9^. This can lead to autonomic and/or neuroendocrine dysfunction, which has been observed across the entire severity spectrum of TBI^10–16^. Indeed, our group and others have utilized indices of heart rate variability to identify ANS dysfunction in the acute and subacute periods following SRC, potentially through disruption of parasympathetic activity^12,13^. With respect to neuroendocrine function, chronic pituitary dysfunction is the most frequently observed abnormality following mTBI; up to 37% of patients show evidence of hypopituitarism at one to five years post-injury, typically characterized by growth hormone deficiency^14^. In addition, pituitary abnormalities have also been observed chronically in sports associated with repetitive head impacts such as football and combat sports^17–19^.

While neuroendocrine abnormalities are often identified in the months to years following TBI, perturbations in the acute and subacute stages have also been reported. It has been suggested that women who suffer an mTBI in the luteal phase of their menstrual cycle, when progesterone is highest, may suffer from subsequent progesterone withdrawal leading to lower neurological outcomes and quality of life at one month post injury^20^. Furthermore, prior observations of elevated blood levels of thyroid stimulating hormone (TSH) and triodothyronine within 48 h of an mTBI suggest that the hypothalamic-pituitary-thyroid axis may also be disrupted acutely after injury In view of this, Merchant-Borna and colleagues^21^ found perturbations in peripheral messenger RNA related to the hypothalamic-pituitary-adrenal (HPA) axis in the subacute phase (7 days) after sport concussion. Similarly, a case series study of four football players sampled after injury showed that blood prolactin levels were acutely suppressed and subsequently increased throughout recovery^22^. And finally, in a study of 14 youth hockey players with SRC, abnormally low (<200 nmol/L) subacute blood cortisol levels were associated with increased symptom reporting and time to recovery in a subset of four athletes^23^.

While evidence of perturbations to both the neuroendocrine and ANS systems have been observed in the acute and subacute period following SRC, it is unclear how this correlates with clinical indices such as symptoms and recovery time. This is particularly relevant to the study of neuroendocrine hormones, given their relationship to a litany of physical and psychological disorders and their profound influence on somatic and behavioural symptomology^24^. Greater knowledge of these relationships in SRC may not only advance our understanding of concussion pathophysiology but may also aid in the development and implementation of therapies that augment the neuroendocrine and ANS systems, such as exercise, mindfulness training, and biofeedback^25^.

Hence, the purpose of this study was to evaluate a panel of neuroendocrine hormones in relation to symptom reporting and recovery in a group of interuniversity athletes within the first week following SRC. We hypothesized that greater symptom burden and longer recovery times would correlate with blood concentrations of neuroendocrine hormones.

## Results

### Participant characteristics

**Figure 1:**
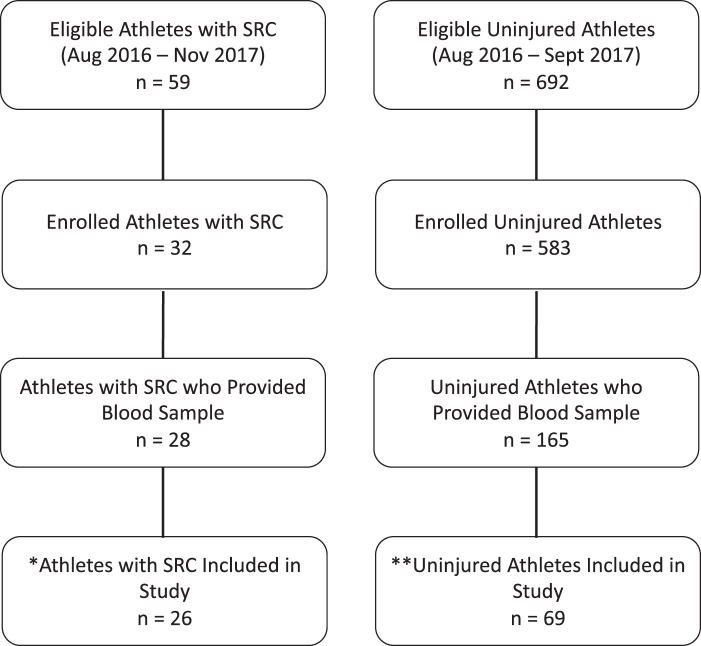
Athlete enrollment from objective measures of sport concussion project, and study participant selection. *One subject removed due to missing symptoms, and one subject removed due to missing values in five of seven hormones. **Thirteen subjects were removed due to subsequent concussion and recruitment in the SRC group; 28 subjects were excluded because they had their blood drawn prior to 11am; 45 subjects were removed because they had exercised within 24 h; 10 subjects were excluded due to repeated enrollment in subsequent years (only a single enrolment per subject was allowed).

Athlete characteristics are described in Table 1. There were no significant differences in age and time from last concussion between healthy athletes and athletes with SRC. Athletes with SRC reported a significantly greater number of symptoms (p < 0.001) and symptom severity (p < 0.001) compared to healthy athletes. The greatest mean differences in symptom clusters between athletes with SRC and healthy athletes were observed for cognitive (median = 6 (SRC) vs. 0 (healthy), bootstrap ratio (BSR) 9.7, p < 0.001) and somatic (median = 12 (SRC) vs. 0 (healthy), BSR = 8.2, p < 0.001) symptoms. No athletes with SRC reported loss of consciousness. For a complete description of athlete enrolment and selection, please see both Fig. 1 and the participants section of the methods.

**Table 1:** Athlete characteristics.

Variable	SRC (n = 26)	Healthy (n = 69)
Age (years)	21.4 (19.4–22.1)	20.3 (19.3–22.2)
Sex (n, % male)	13 (50.0)	43 (62.3)
Sport (n, %)		
Baseball	—	1 (1.4)
Basketball	1 (3.8)	6 (8.7)
Field Hockey	1 (3.8)	4 (5.8)
Football	3 (11.5)	14 (20.3)
Ice Hockey	7 (26.9)	11 (15.9)
Lacrosse	4 (15.4)	9 (13.0)
Mountain Biking	1 (3.8)	—
Rugby	7 (26.9)	5 (7.2)
Soccer	1 (3.8)	16 (23.2)
Volleyball	1 (3.8)	2 (2.9)
Water Polo	—	1 (1.4)
Total Symptoms	10.0 (8.0–15.0)	2.0.(0–5.0)
Symptom Severity	19.0 (11.0–32.0)	2.0 (0–6.0)
Days to blood sample from injury	3.5 (range = 2–7)	—
Days to medical clearance	25.0 (16.0–60.0)	—
Concussion History (n, %)	13 (50.0)	24 (34.8)
Time since last concussion (years)	2.8 (1.7–4.5)	4.1 (3.0–6.1)

sport-related concussion (SRC).

All values reported as the median and interquartile range, unless otherwise stated.

### Hormone profiles between athletes with SRC and healthy athletes

Hormone concentrations in athletes with SRC and healthy athletes can be seen in Table 2. PLSDA analysis of hormone profiles yielded no significant differences between groups.

**Table 2:** Hormone concentrations in athletes.

Hormone	Healthy athletes (N, T = 69; M = 43; F = 26)	Athletes with SRC (N, T = 26; M = 13; F = 13)	P	FDR
ACTH (pg/mL)	T = 24.5 (20.5–32.2) M = 25.7 (21.8–32.2) F = 23.2 (19.5–32.6)	T = 27.1 (19.8–36.7) M = 29.0 (21.7–36.7) F = 25.1 (19.7–31.6)	0.631	No
Cortisol (nmol/L)	T = 306.0 (233.0–403.0) M = 306.0 (245.5–385.0) F = 310.5 (212.5–409.0)	T = 285.5 (240.2–346.0) M = 270.0 (227.0–298.0) F = 290.0 (262.0–350.0)	0.307	No
DHEA-S (μmol/L)	T = 8.1 (5.4–10.3) M = 9.1 (6.7–11.2) F = 5.4 (4.3–9.1)	T = 8.2 (7.2–11.0) M = 10.6 (6.9–11.8) F = 7.9 (7.3–9.0)	0.218	No
Prolactin (mIU/L)	T = 167.0 (136.8–218.2) M = 162.0 (127.5–206.5) F = 189.0 (152.0–231.0)	T = 187.0 (134.0–237.0) M = 225.0 (146.0–242.0) F = 171.0 (119.5–197.0)	0.761	No
Progesterone (nmol/L)	T = 1.5 (1.2–2.0) M = 1.5 (1.3–1.9) F = 1.5 (1.1–3.2)	T = 1.6 (1.0–2.7) M = 1.4 (1.0–1.8) F = 2.5 (1.1–11.8)	0.978	No
FT4 (pmol/L)	T = 14.7 (13.6–15.8) M = 14.7 (13.9–16.1) F = 14.8 (13.4–15.7)	T = 14.2 (13.2–16.0) M = 15.3 (14.0–16.3) F = 13.6 (11.3–14.8)	0.356	No
TSH (μIU/L)	T = 1.5 (1.1–2.1) M = 1.6 (1.2–2.3) F = 1.1 (0.9–1.8)	T = 1.6 (1.0–2.2) M = 2.2 (1.2–2.6) F = 1.4 (0.8–1.6)	0.485	No

SRC, sport-related concussion; FDR, false discovery rate; T, total; M, male; F, female; ACTH, adrenocorticotropic hormone; DHEA-S, dehydroepiandrosterone sulfate; FT4, free thyroxine; TSH, thyroid stimulating hormone.

Hormone values presented as the median and interquartile range.

Group differences derived via PLSDA on sex-adjusted z-scores, empirical p-values derived from bootstrapped ratios (mean/SD) over 5000 iterations, corrected at an FDR of 0.05.

### Correlation between hormone profiles and days to medical clearance in athletes with SRC

**Figure 2:**
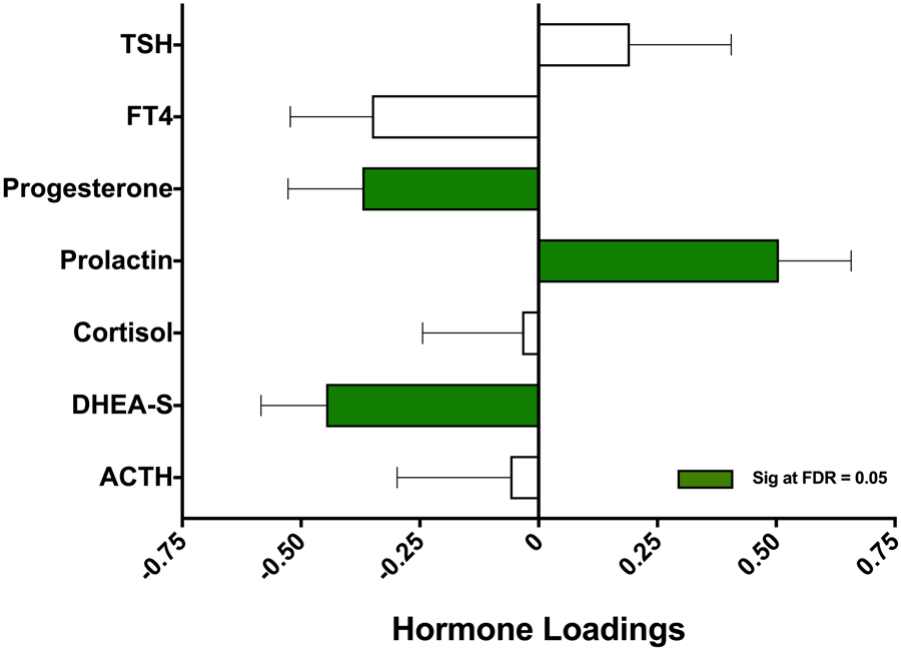
Correlation between neuroendocrine hormones and days to medical clearance. Thyroid stimulating hormone (TSH), free thyroxine (FT4), dehydroepiandrosterone sulfate (DHEA-S), and adrenocorticotropic Hormone (ACTH). Plot shows the correlations between neuroendocrine hormones measured in the subacute period following sport-related concussion (SRC) and days to medical clearance by partial least squares (PLS) analysis. Bars represent biomarker loadings and standard errors derived from bootstrapped resampling (5000 samples). Green bars = significant at a false discovery rate (FDR) < 0.05.

PLS analysis of the relationship between days to medical clearance and blood hormone concentrations in athletes following SRC can be seen in Fig. 2. A significant positive relationship between days to medical clearance and prolactin concentrations was observed (BSR = 3.3, p < 0.001). In addition, a significant negative relationship was found between days to medical clearance and both progesterone (BSR = 2.4, p = 0.02) and DHEA-S (BSR = 3.3, p = 0.001).

### Correlation between hormone profiles and symptoms in athletes with SRC

**Figure 3:**
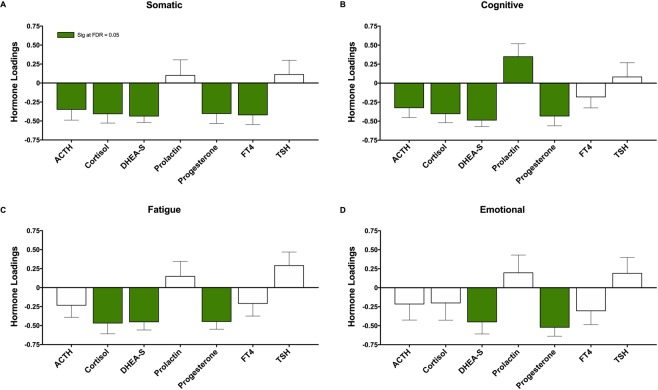
Correlation between neuroendocrine hormones and symptom clusters. Adrenocorticotropic Hormone (ACTH), (DHEA-S), free thyroxine (FT4), thyroid stimulating hormone (TSH). Plots show the correlations between neuroendocrine hormones measured in the subacute period following sport-related concussion and (**A)** somatic symptoms, (**B)** cognitive symptoms, (**C)** fatigue symptoms, and (**D)** emotion symptoms, by partial least squares (PLS) analysis. Bars represent biomarker loadings and standard errors derived from bootstrapped resampling (5000 samples). Green bars = significant correlation with days to recovery at a false discovery rate (FDR) <0.05.

A plot of the PLS analyses evaluating the relationship between symptoms clusters and blood hormone levels can be found in Fig. 3. The somatic symptom cluster in athletes with SRC was negatively correlated with blood concentrations of all hormones excluding prolactin and TSH, with the greatest effects seen for DHEA-S (BSR = 5.5, p < 0.001) (Fig. 3A).

The cognitive symptom cluster was significantly associated with all blood hormone concentrations excluding FT4 and TSH; symptoms were negatively correlated with ACTH, cortisol, DHEA-S and progesterone, with DHEA-S showing the greatest effects (BSR = 6.1, p < 0.001) (Fig. 3B). In addition, the cognitive cluster was the only symptom cluster positively associated with prolactin concentrations (BSR = 2.1, p = 0.03) (Fig. 3B).

The fatigue symptom cluster was negatively correlated with cortisol (BSR = 3.4, p < 0.001), DHEA-S (BSR = 4.4, p < 0.001) and progesterone (BSR = 4.6, p < 0.001) (Fig. 3C). Finally, we observed a significant negative relationship between symptoms of emotion and both DHEA-S (BSR = 2.9, p = 0.003) and progesterone (BSR = 4.7, p < 0.001) (Fig. 3D).

## Discussion

The main finding of the current study was that greater symptom burden and longer time to medical clearance were correlated with neuroendocrine hormones measured in the blood of athletes following SRC. Importantly, we did not find group-level differences in neuroendocrine hormones between athletes with SRC and healthy athletes, suggesting that perturbations to the neuroendocrine system may not be a ubiquitous biological consequence of concussion but exist only in those with unfavorable clinical profiles.

We observed a negative relationship between days to medical clearance and blood concentrations of both DHEA-S and progesterone in athletes with SRC. Moreover, both hormones were negatively corelated with all self-reported symptom clusters (somatic, cognitive, fatigue, emotion). That lower levels of DHEA-S and progesterone were related to increased symptom burden and longer recovery times is consistent with a large body of animal and human evidence which has identified a potential therapeutic benefit of DHEA-S and progesterone following moderate and severe TBI^26–32^. DHEA-S is as a pro-excitatory neurosteroid that may alleviate glutamate excitotoxicity^33,34^, promote dendrite arborization^35^, and increase cholinergic neurotransmission^36^. In rodent models of mild-to-moderate TBI, DHEA-S treatment has been shown to improve behavioural recovery in sensorimotor and cognitive tasks following injury^29–31^.

While progesterone is derived from a common precursor to DHEA-S, pregnenolone, the neurophysiologic effects of progesterone differ from that of DHEA-S. Yet, progesterone has also been considered as a potential therapeutic target for TBI^26–28,32^ as it may reduce cerebral edema and blood brain barrier disruption, as well as limit oxidative stress and inflammation post-injury^37–40^. In humans, stage II clinical trials have shown potential for progesterone as a neuroprotective agent following moderate and severe TBI^41,42^. Although therapeutic benefits were not observed in Stage III trials^43,44^, this isn’t surprising given the heterogeneity and complexity of TBI, and thus it is still likely that progesterone is involved in injury pathophysiology. Taken together, our results are consistent with the body of literature that suggests both DHEA-S and progesterone influence recovery and symptomology following concussion.

In addition to DHEA-S, we observed a negative relationship between ACTH, cortisol and somatic, cognitive and fatigue (cortisol only) symptom clusters. These results are supported by the findings of Merchant-Borna and colleagues^21^ who observed a downregulation of a number of HPA-axis genes in the peripheral blood mononuclear cells of athletes measured seven days following SRC, and are also in-line with those of Ritchie and colleagues^23^, who found that lower cortisol levels in pediatric sport concussion were related with longer recovery and greater symptom burden. While our group-level analysis did not identify differences in hormone concentrations between athletes with SRC and healthy athletes, our correlational analyses highlight that these hormones may only be lower in a subset of injured athletes experiencing greater symptom burden. We also observed that greater somatic symptoms correlated with lower concentrations of the hypothalamic-pituitary-thyroid (HPT) axis hormone FT4. In view of this, Cernak and colleagues^45^ identified elevated blood concentrations of TSH acutely following mTBI. While we did not find any significant group differences or correlations with TSH concentrations, and did not clinically evaluate athletes for the presence of thyroid dysfunction, our data, along with that of Cernak and colleagues, is consistent with the direction of TSH and FT4 levels found in the blood of patients with overt hypothyroidism^46^, and suggests that dysfunction of the HPT axis may be present in a subset of concussed individuals presenting with higher somatic symptoms.

We observed that higher symptoms of emotion were associated with lower progesterone and DHEA-S concentrations. Interestingly, both progesterone and DHEA-S have been correlated with improvements to mood and behavior^47–50^. While the underlying mechanisms are not fully understood, these results justify future research into the role of these hormones in both injury-related affect and the potential development of mental health issues following concussion; it has been theorized that disruption to the neuroendocrine system may play an important mediating role^51^.

We observed a positive correlation between blood concentrations of prolactin and both days to medical clearance and cognitive symptom reporting. These findings are consistent with severe TBI studies where prolactin levels are often elevated following injury and may infer damage to the hypothalamus^52–54^. However, our findings differ from the only other known related study in sport concussion; in a case series of four male football players, it was observed that concussion recovery was associated with suppressed prolactin levels that increased throughout recovery^22^. Yet, prolactin is the most biologically diverse pituitary hormone, with numerous functions including the modulation of metabolism, immunity, behavior, lactation, and reproduction^55^. Our understanding of the role of prolactin in the sequelae of sport concussion remains limited, however, our findings warrant further investigation.

The summation of the results in the present study are consistent with other facets of secondary injury previously observed following SRC by our group, namely oxidative stress^56^, and inflammation^57,58^. Specifically, we have previously identified higher concentrations of the oxidative stress marker PRDX-6 in athletes following SRC^56^, and also  found that days to medical clearance was positively correlated with blood concentrations of the inflammatory chemokines monocyte chemoattractant protein (MCP)-1 and MCP-4^59^. In the current study, greater symptom burden and recovery time were associated with lower DHEA-S and progesterone; progesterone may limit oxidative stress following TBI, while DHEA-S and progesterone have both been associated with reducing neuroinflammation^30,37^. In addition, we observed that higher levels of prolactin - a potential proinflammatory mediator -  were related to increased time to recovery and cognitive symptom reporting^60,61^. Hence, the relationship between an unfavorable clinical profile (symptom reporting and time to medical clearance), lower levels of hormones theorized to reduce inflammation and oxidative stress (DHEA-S and progesterone), and higher levels of a proinflammatory-associated hormone (prolactin) are consistent with our previous works and support a role for the neuroendocrine system in multiple facets of secondary injury.

There were several limitations in the current study that must be considered when interpreting the results. First, while we controlled for the effects of sex, a larger sample size would have allowed for separate male and female analyses, including controlling for menstrual cycle phase and birth control use in female athletes. Furthermore, the absence of morning fasting blood samples precluded clinical quantitation of patients as “normal” or “abnormal” for individual hormone concentrations. In order to maximize participant recruitment while avoiding diurnal variation, we chose a sampling period between 11:00am and 6:00 pm. Specifically, this allowed us to avoid the typical morning peaks seen with hormones such as prolactin, cortisol and progesterone^62–64^. In addition, we measured DHEA-S, which displays less diurnal variation than DHEA^64,65^, implemented a partial regression to eliminate the statistical variance caused by changes in hormone concentrations related to the time of day of blood sampling, and evaluated the potential relationship between hormones and the time from injury to blood sampling (no significant correlations found by PLS; data not shown). Yet, a narrower sample timing may still have been helpful in reducing biological noise. Lastly, while uninjured athletes in the current study were recruited from a potential pool of 11 sports selected *a priori* due to their high risk for concussion, our institution enrolls concussions from athletes participating in any sport. Hence, this creates a potential risk for selection bias. However, we do not feel that this significantly impacted our results as they pertain to concussion; while sports varied within and between groups, our cohort had adequate representation from sport types characterized by no contact, incidental contact, and purposeful contact/collision. In addition, while it is  theoretically possible that the athletes within the SRC group may differ from those who did not agree to participate, both the medical time to clearance and symptom burden found in the current study are in-line with previous studies by our group using different samples from an athlete population^3,59,66^.

Neuroendocrine hormones measured in the peripheral blood following SRC correlate with higher symptom reporting and longer time to medical clearance. Specifically, an unfavorable clinical profile correlates with a blood hormone signature that is characterized by lower HPA-axis activity, lower progesterone, and elevated prolactin. Future research is warranted to elucidate the role of the hypothalamic-pituitary axes in concussion pathophysiology, and its potential involvement with other secondary injury processes such as inflammation and oxidative stress.

## Methods

### Participants

Participant eligibility and enrolment information can be seen in Fig. 1. From August 2016 – November 2017, 692 uninjured athletes and 59 athletes with an SRC were eligible for participation in the current study as part of a larger multi-year study being conducted at a single academic institution. Of this cohort, 583 uninjured athletes were enrolled prior to the start of their competitive season, and 32 athletes with SRC were enrolled at the time of their injury: blood was taken from 165 uninjured athletes during preseason baseline testing and from 28 athletes within seven days of an SRC. Due to an absence of symptom or hormone data, two athletes were excluded from the SRC group. In addition, 96 athletes were excluded from the uninjured cohort; 13 healthy athletes suffered a concussion and were therefore moved to the SRC group, 28 were removed due to blood draws prior to 11am, 45 were removed due to exercising within 24 h of blood sampling, and 10 athletes were removed due to having multiple samples. Hence, a final sample of 95 athletes (male = 56, female = 39) from 11 interuniveristy sports were enrolled, comprising 69 uninjured athletes and 26 athletes with an SRC (Table 1**)**. Concussion diagnosis was performed by a sport medicine physician at an academic sport medicine clinic, in accordance with the Concussion in Sport Group statement^2^. Medical clearance for unrestricted activity (i.e., clinical recovery) was determined by a physician with expertise in sport and exercise medicine from a single institution. Specifically, clinical recovery required (1) athletes to be asymptomatic at rest, and (2) completion of a graded return-to-play (RTP) protocol. RTP stages included: light aerobic exercise, more intensive training, sports-specific exercises, non-contact participation (i.e., low risk practice), and high-risk practice. Finally, following the completion of RTP stages, the physician ensured a return to their baseline level of cognitive functioning and neurological functioning. Exclusion criteria for all study participants included a history of SRC within six months of study participation, a history of neuroendocrine disorders, exercise within 24 h of enrollment, or blood sampling prior to 11am. All study participants provided written informed consent prior to enrollment, and all study procedures were in accordance of the declaration of Helsinki, and approved by the Health Sciences Research Ethics Board, University of Toronto (protocol reference # 27958).

### Blood sampling

Blood was sampled from athletes with an SRC within seven days of injury (median = 3.5, range = 2–7), while uninjured athletes were sampled at an assessment prior to their competitive season. All samples were taken between 11:00am and 6:00 pm. Blood was not taken from athletes who presented with a known acute infection or illness at the time of sampling or were taking any medications beyond birth control. Venous blood was drawn into 10-mL K_2_EDTA and 4-mL serum vacutainers (Becton Dickinson, NJ, USA), where it equilibrated for approx. one hour at room temperature, followed by a two min centrifugation using a PlasmaPrep 12^TM^ centrifuge (Separation Technology Inc., FL, USA). The supernatant was then aliquoted and frozen at −70 °C until analysis.

### Hormone analysis

Blood concentrations of seven hormones were quantitated by solid-phase chemiluminescent immunoassay via the Immulite 1000 analyzer (Siemens Healthineers Global, Erlangen, Germany). Adrenocorticotropic hormone (ACTH) was run on plasma supernatant, while serum supernatant was used for the remaining six hormones: cortisol, dehydroepiandrosterone sulfate (DHEA-S), free thyroxine (FT4), progesterone, prolactin, and thyroid stimulating hormone (TSH).

### Symptom reporting

The Post-Concussion Symptom Scale (PCSS) was administered to all athletes at the time of blood draw. The PCSS is a 22-item symptom inventory checklist using a seven-point likert scale rating included in the most recent Sport Concussion Assessment Tool version 5 (SCAT-5)^67^. Symptoms were clustered into four categories for statistical analysis: somatic (headache, pressure in head, neck pain, nausea/vomiting, dizziness, blurred vision, balance problems, sensitivity to light, sensitivity to noise), cognitive (feeling slowed down, feeling “in a fog”, “don’t feel right”, difficulty concentrating, difficulty remembering, confusion), fatigue and sleep problems (fatigue/low energy, drowsiness, trouble falling asleep) and emotion (more emotional, irritability, sadness, nervous/anxious)^68^.

### Statistical analysis

Hormones were statistically quantitated if they fell within the assay detection range as stated by the manufacturer: ACTH (9–1250 pg/mL), Cortisol (28–1380 nmol/L), DHEA-S (0.41–27μmol/L), progesterone (0.64–127 nmol/L), prolactin (11–3180 mIU/L), TSH (0.004–75 μIU/L), and FT4 (3.9–77.2 pmol/L). One participant for each of ACTH and TSH had a single value below the assay detection limit; these values were imputed with ½ the lower limit of detection. Two prolactin values were >5 standard deviations (SDs) above the mean, and one TSH value was >7 SDs above the mean; these values were deemed outliers and were removed and imputed with the respective median hormone values from the appropriate group.

Prior to statistical analysis and following outlier removal and imputation, hormone values were checked for violations of normality. Skewness and kurtosis for SRC and healthy hormone values were separately evaluated against 1000 resamples of a random normal gaussian model. In athletes with SRC, skewness ranged from −1.8 (p < 0.001) to 2.4 (p < 0.001), and kurtosis ranged from 2.9 (p = 0.799) to 8.5 (p = 0.001). In uninjured athletes, skewness ranged from −1.1 (p < 0.002) to 5.3 (p < 0.001), and kurtosis ranged from 3.7 (p = 0.161) to 34.8 (p < 0.001). Hence, all data was rank transformed prior to statistical evaluation.

Between-group comparisons (SRC vs. healthy) of athlete characteristics (age, total symptoms, symptom severity, somatic, cognitive, fatigue and emotion symptoms) were calculated by bootstrap resampling (5000 iterations) in order to generate a bootstrap ratio (BSR) (effect size; mean/standard deviation (SD)) of the mean difference. From this, an empirical p value was obtained and corrected at a false discovery rate (FDR) threshold of 0.05.

Prior to multivariate analysis, A partial regression was implemented to remove the confounding influence of time-of-day of sampling on blood hormone concentrations. For each hormone, separate ordinary least squares regression tests were run on rank-transformed data against time of day of blood sampling. The residual values of each test were then used as the adjusted hormone values for further analysis.

Hormone profiles between athletes with SRC and healthy athletes were analyzed by partial least squares discriminant analysis (PLSDA)^69^. PLSDA is a multivariate tool used to maximize the covariance between a set of predictor variables (hormones) and a binary response variable (SRC vs. healthy). The PLSDA was run in a bootstrap resampled framework (5000 iterations), followed by the generation of BSRs and empirical p values, corrected at an FDR of 0.05. Due to the difference in hormone profiles between males and females (data not shown), prior to PLSDA, rank-normalized hormone values were transformed into z-scores created separately on male and female participants.

A correlational PLS (PLSC) was used to evaluate the relationship between hormones and (1) symptom clusters and (2) days to physician medical clearance. PLSC is similar to PLSDA, but allows the evaluation of continuous response variables^69^. Prior to PLSC analysis, hormone z-scores were generated separately for male and female athletes with SRC using the mean of the hormone values in their respective healthy athlete groups. For PLS plots, hormones are represented by the mean and standard error of the bootstrapped loadings (Figs. 1 and 2).

## Data Availability

All data is available upon request due to privacy restrictions.
